# Network Pharmacology-Based Analysis on the Potential Biological Mechanisms of Sinisan Against Non-Alcoholic Fatty Liver Disease

**DOI:** 10.3389/fphar.2021.693701

**Published:** 2021-08-27

**Authors:** Xiaoyi Wei, Weixin Hou, Jiajun Liang, Peng Fang, Bo Dou, Zisong Wang, Jiayang Sai, Tian Xu, Chongyang Ma, Qiuyun Zhang, Fafeng Cheng, Xueqian Wang, Qingguo Wang

**Affiliations:** ^1^ School of Traditional Chinese Medicine, Capital Medical University, Beijing, China; ^2^ Department of Traditional Chinese Medicine, Beijing Chaoyang Hospital, Capital Medical University, Beijing, China; ^3^ Department of Oncology, The Third Affiliated Hospital, Beijing University of Chinese Medicine, Beijing, China; ^4^ School of Traditional Chinese Medicine, Beijing University of Chinese Medicine, Beijing, China

**Keywords:** network pharmacology, NAFLD, Sinisan, protein-protein interaction (PPI) network, topological analysis, JAK2/STAT3

## Abstract

Non-alcoholic fatty liver disease (NAFLD) has become the most prevalent liver disease in China. Sinisan (SNS) is a traditional Chinese medicine formula that has been widely used in treating chronic liver diseases, including NAFLD. However, its underlying biological mechanisms are still unclear. In this study, we employed a network pharmacology approach consisting of overlapped terms- (genes or pathway terms-) based analysis, protein-protein interaction (PPI) network-based analysis, and PPI clusters identification. Unlike the previous network pharmacology study, we used the shortest path length-based network proximity algorithm to evaluate the efficacy of SNS against NAFLD. And we also used random walk with restart (RWR) algorithm and Community Cluster (Glay) algorithm to identify important targets and clusters. The screening results showed that the mean shortest path length between genes of SNS and NAFLD was significantly smaller than degree-matched random ones. Six PPI clusters were identified and ten hub targets were obtained, including STAT3, CTNNB1, MAPK1, MAPK3, AGT, NQO1, TOP2A, FDFT1, ALDH4A1, and KCNH2. The experimental study indicated that SNS reduced hyperlipidemia, liver steatosis, and inflammation. Most importantly, JAK2/STAT3 signal was inhibited by SNS treatment and was recognized as the most important signal considering the network pharmacology part. This study provides a systems perspective to study the relationship between Chinese medicines and diseases and helps to discover potential mechanisms by which SNS ameliorates NAFLD.

## Introduction

Non-alcoholic fatty liver disease (NAFLD) is the most common liver disease with a global prevalence of 25% (Cotter and Rinella, 2020). Although NAFLD was traditionally considered as a benign condition and only patients with obesity had been more likely to receive medical care, recent evidence has shown it to be a far more complex disease. Increased NAFLD-related mortality has been observed in several studies from the past years (Allen et al., 2019; Targher et al., 2020). And even simple steatosis might finally result in increased all-cause mortality because of the progression of this disease after a follow-up observation over decades (Tilg and Targher, 2020). NAFLD includes various clinical phenotypes ranging from simple steatosis (a benign condition called fatty liver) to non-alcoholic steatohepatitis (NASH) and hepatic fibrosis. And these conditions increase the risks of liver-related complications, such as cirrhosis, liver failure, and hepatocellular carcinoma. NAFLD is also closely associated with important extra-hepatic manifestations, such as cardiovascular disease and chronic kidney disease, which further increases its disease burden. Therefore, it has to be acknowledged that control of this disease would have a major impact on the benefits of health care.

Sinisan (SNS), also called Shigyaku-san in Japan, is a classic Chinese medicine formula originating from the book of Treatise on Febrile Diseases (Shang Han Lun) of the Han Dynasty (200–201 AD) China. It comprises four botanical drugs: *Bupleurum chinense DC.* (Apiaceae*; Bupleuri radix*) (Chaihu), *Paeonia lactiflora Pall.* (Paeoniaceae*; Paeoniae radix alba*) (Shaoyao), *Gardenia jasminoides J. Ellis* (Rubiaceae*; Aurantii fructus immaturus*) (Zhishi), and *Glycyrrhiza uralensis Fisch.* (Leguminosae*; Glycyrrhizae radix et rhizoma*) (Gancao) with a dose proportion of 1:1:1:1. For centuries, SNS has been widely applied in treatment of chronic liver diseases in the clinic. Our previous study indicated that SNS decreased liver steatosis and inflammation in NAFLD rats (Mu et al., 2020). However, for the well-accepted multi-component and multi-target effects of TCM formulas, it is difficult to understand the potential biological mechanisms by the traditional pharmacology approach. Network pharmacology was a recently proposed TCM research strategy to use a “network target” as a mathematical and computable representation of various connections between botanical formulae and diseases (Li and Zhang, 2013; Li et al., 2014). Therefore, we used this system biology-based approach to describe the association of multiple components with multiple targets and multiple pathways and recovery of the potential mechanisms of SNS against NAFLD at a system level.

**FIGURE 1:**
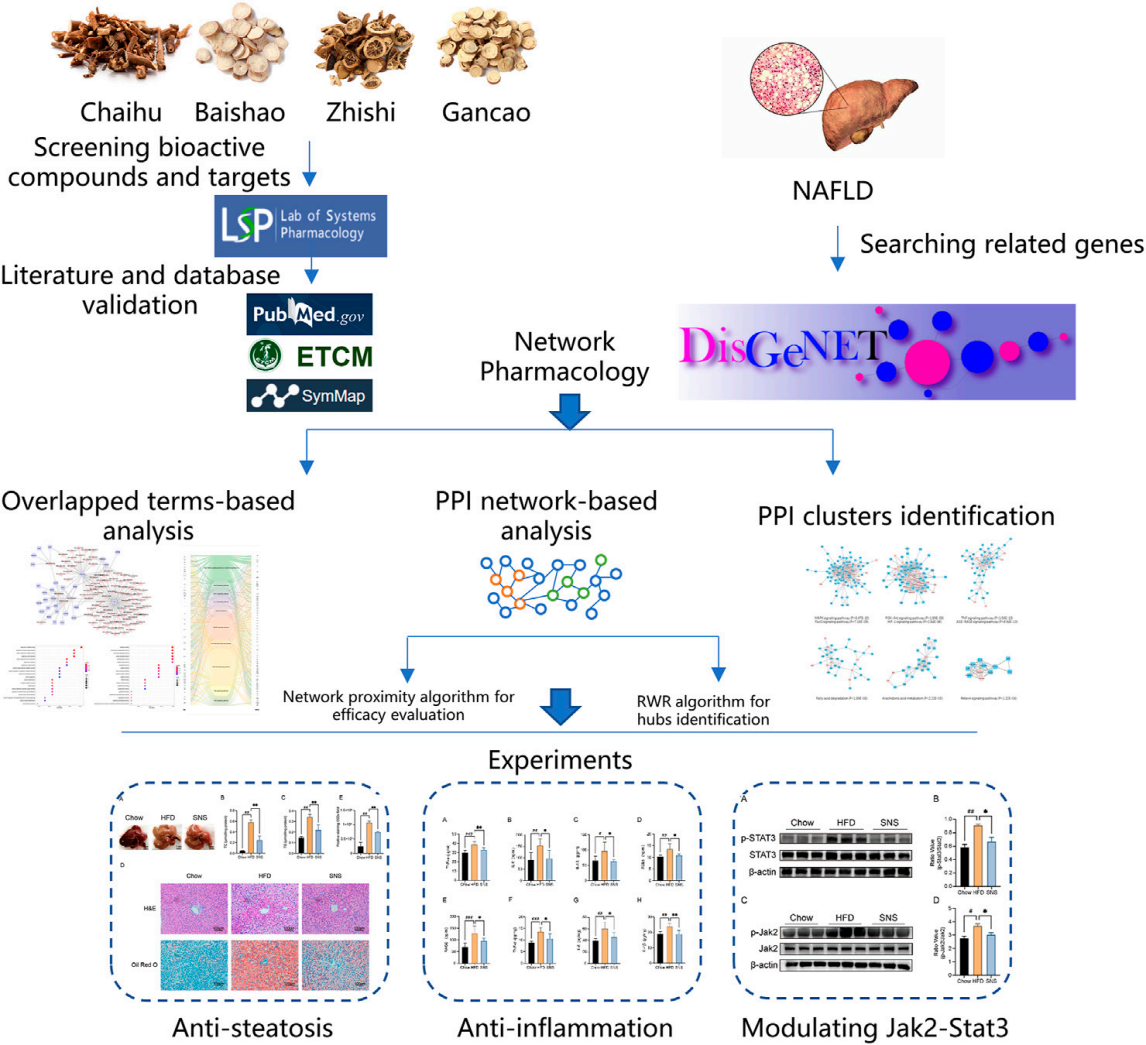
Integrated workflow for discovery of the potential mechanisms of SNS against NAFLD.

In the present work, we, firstly, investigated the effect of SNS against NAFLD in high fat diet- (HFD-) induced NAFLD rat model. Next, we collected information about compounds, compound-related targets, and NAFLD-related genes from extensive databases and identified important targets and pathways using a protein-protein interaction network-based method. Finally, a series of *in vivo* experiments were conducted to validate important targets of SNS against NAFLD (Figure 1).

## Methods

### Collection of Bioactive Compounds and Prediction of Corresponding Targets

All compounds of SNS decoction and their corresponding absorption, distribution, metabolism, and excretion (ADME) parameters were obtained from the Traditional Chinese Medicine Systems Pharmacology Database and Analysis Platform (TCMSP). OB value (systemic bioavailability after oral absorption and distribution) ≥30% and DL value (structural similarity between compounds and clinically used drugs in the DrugBank database) ≥ 0.18 were employed as criteria to filter bioactive compounds. A total of 137 distinct bioactive compounds with literature or other database validation were identified for further analysis (Table 1). The putative targets of bioactive compounds in SNS decoction were predicted using TCMSP database as we previously mentioned. After removing duplicate genes, we obtained 155 SNS targets for further analysis.

**TABLE 1:** Top 10 genes ranked by random walk with start algorithm.

Rank	Gene	Score
1	STAT3	0.005377152
2	CTNNB1	0.004535756
3	MAPK1	0.004386918
4	MAPK3	0.004010912
5	AGT	0.003464108
6	NQO1	0.002783255
7	TOP2A	0.002570216
8	FDFT1	0.00255187
9	ALDH4A1	0.002469136
10	KCNH2	0.002469136

### Prediction of Non-Alcoholic Fatty Liver Disease Genes

Disease genes were obtained using the recently updated DisGeNET database with keywords “Non-alcoholic Fatty Liver Disease”, “FATTY LIVER DISEASE, NONALCOHOLIC”, “Nonalcoholic Steatohepatitis”, and “Fibrosis, Liver” (Piñero et al., 2020). After removing BEFREE text mining genes, we obtained 306 unduplicated genes related to NAFLD and the details are provided in Table 2.

**TABLE 2:** Identified bioactive compounds in SNS from UPLC-ESI-MS/MS Analysis.

ID	Name	Status	Ion mode
MOL000098	Quercetin	Confirmed	Positive
MOL000211	Mairin	Confirmed	Negative
MOL000239	Jaranol	Confirmed	Negative
MOL000354	Isorhamnetin	Confirmed	Negative
MOL000359	Sitosterol	Confirmed	Positive
MOL000392	Formononetin	Confirmed	Negative
MOL000417	Calycosin	Confirmed	Positive
MOL000422	Kaempferol	Confirmed	Negative
MOL000497	Licochalcone A	Confirmed	Positive
MOL000500	Vestitol	Confirmed	Negative
MOL001484	Inermine	Confirmed	Negative
MOL001792	DFV	Confirmed	Positive
MOL002311	Glycyrol	Confirmed	Positive
MOL002565	Medicarpin	Confirmed	Negative
MOL004328	Naringenin	Confirmed	Positive
MOL004841	Licochalcone B	Confirmed	Positive
MOL004848	Licochalcone G	Confirmed	Positive
MOL004855	Licoricone	Confirmed	Positive
MOL004879	Glycyrin	Confirmed	Positive
MOL004883	Licoisoflavone	Confirmed	Positive
MOL004884	Licoisoflavone B	Confirmed	Negative
MOL004885	Licoisoflavanone	Confirmed	Positive
MOL004908	Glabridin	Confirmed	Positive
MOL004910	Glabranin	Confirmed	Positive
MOL004911	Glabrene	Confirmed	Negative
MOL004912	Glabrone	Confirmed	Positive
MOL004915	Eurycarpin A	Confirmed	Positive
MOL004917	Glycyroside	Confirmed	Positive
MOL004948	Isoglycyrol	Confirmed	Negative
MOL004949	Isolicoflavonol	Confirmed	Positive
MOL004957	HMO	Confirmed	Positive
MOL004961	Quercetin der	Confirmed	Negative
MOL005000	Gancaonin G	Confirmed	Negative
MOL005020	Dehydroglyasperins C	Confirmed	Negative
MOL002776	Baicalin	Confirmed	Positive
MOL004609	Areapillin	Confirmed	Positive
MOL004702	Saikosaponin c_qt	Confirmed	Negative
MOL013187	Cubebin	Confirmed	Negative
MOL000492	(+)-Catechin	Confirmed	Negative
MOL001918	Paeoniflorgenone	Confirmed	Positive
MOL001921	Lactiflorin	Confirmed	Positive
MOL001924	Paeoniflorin	Confirmed	Negative
MOL001925	paeoniflorin_qt	Confirmed	Negative
MOL001928	albiflorin_qt	Confirmed	Positive
MOL001930	Benzoyl paeoniflorin	Confirmed	Positive
MOL000006	Luteolin	Confirmed	Positive
MOL001798	neohesperidin_qt	Confirmed	Negative
MOL001803	Sinensetin	Confirmed	Positive
MOL001941	Ammidin	Confirmed	Positive
MOL005100	5,7-Dihydroxy-2-(3-hydroxy-4-methoxyphenyl)chroman-4-one	Confirmed	Negative
MOL005828	Nobiletin	Confirmed	Positive
MOL005849	Didymin	Confirmed	Negative
MOL007879	Tetramethoxyluteolin	Confirmed	Positive
MOL013276	Poncirin	Confirmed	Negative
MOL013277	Isosinensetin	Confirmed	Positive
MOL013279	5,7,4′-Trimethylapigenin	Confirmed	Positive
MOL013352	Obacunone	Confirmed	Negative
MOL013428	Isosakuranetin-7-rutinoside	Confirmed	Negative

### Construction of Protein-Protein Interaction Networks

We used two highly cited human protein-protein interaction data for background network constructions, dataset constructed by Professor Barabasi’s team (Cheng et al., 2018) and the Search Tool for the Retrieval of Interacting Genes (STRING) database (Szklarczyk et al., 2019). The first dataset was derived from 15 commonly used databases with experimental evidence and the in-house confirmed data without inferred information. We constructed a PPI network containing 16,677 nodes and 243,603 edges and called it Bnet. The second dataset provided functional associations for proteins, which were sorted by a confidence score. Data only for “*Homo sapiens*” with a confidence score ≥0.9 was used for Snet construction, containing 9,941 nodes and 227,186 edges.

### Network-Based Efficacy Evaluation of Sinisan Against Non-Alcoholic Fatty Liver Disease

Here, we used the network proximity index proposed by Prof. Barabasi’s group to evaluate the efficacy of drugs against diseases in the background of Bnet (Cheng et al., 2019). Specifically, we marked T and V as the set of SNS genes and the set of NAFLD genes, respectively. We defined the shortest path length between nodes v ∈ V and t ∈ T in the network as Equation 1.

$$d_{c}\left(V,T\right)=\frac{1}{\mathrm{\|T\|}}\underset{t\in T}{\sum }min_{v\in V}d\left(v,t\right).$$ (1)

We also compared the shortest path length between genes of SNS and NAFLD with the expected shortest path length between two random and size-matched groups of genes. As shown in Equation 2, we calculated the mean *µ*_d(V,T)_ and standard deviation *s*_d(V,T)_ of the reference distribution and converted the absolute distance d_c_ to a relative distance Z_dc._

$$Z_{d_{c}}=\frac{d_{c}-{\text{μ}}_{d_{c}\left(V,T\right)}}{{\text{σ}}_{d_{c}\left(V,T\right)}}.$$ (2)

### Network-Based Important Genes Identification of Sinisan against Non-Alcoholic Fatty Liver Disease

In this study, the random walk with restart (RWR) algorithm, a classic ranking algorithm, was adopted to select important genes in the sub-network based on Snet. As shown in Equation 3, the vector p_0_ is the initial probability distribution. Therefore, in p_0_, only the seeds have values different from zero. After several iterations, the difference between the vectors p_t+1_ and p_t_ becomes negligible, the stationary probability distribution is reached, and the elements in these vectors represent a proximity measure from every graph node to the seeds. In this work, iterations are repeated until the difference between p_t_ and p_t+1_ falls below 10^–10^ (Valdeolivas et al., 2019).

$$\text{p}\begin{array}{c} T\\ t+1 \end{array}=\left(1-r\right)Mp\begin{array}{c} T\\ t \end{array}+rp\begin{array}{c} T\\ 0 \end{array}.$$ (3)

In detail, before the algorithm was executed, a sub-network of SNS genes and NAFLD genes was obtained from the Snet and considered to be the core PPI network of SNS against NAFLD. The RWR algorithm was employed with seed genes as SNS genes and a restarting probability (r) of 0.75 by RandomWalkRestartMH R package, as in previous studies (Yang et al., 2020). The top 10 genes with the highest affinity scores were identified. The affinity score referred to the proximity between two nodes and the parameter “r” is the probability of moving to seed nodes.

### Network Construction and Enrichment Analysis

Compound-gene networks and PPI networks were constructed by Cytoscape software (Version 3.8). To identify clusters of the sub-PPI network of SNS genes and NAFLD genes, ClusterMaker 2 plugin for Cytoscape was applied by the Community Cluster (Glay) algorithm (Morris et al., 2011). For gene enrichment analysis, gene ontology (GO) enrichment of these genes includes a biological process (BP), molecular function (MF), and cellular component (CC). For comparison between the SNS gene set and NAFLD gene set, the ClusterProfiler package of R 3.5.0 software is adopted to conduct GO enrichment (Yu et al., 2012). Kyoto Encyclopedia of Genes and Genomes (KEGG) pathway enrichment analysis of core targets is also carried out and visualized using the ggplot2 R package. *P* value < 0.05 was set to be significant. A suite of KEGG mapping tools, KEGG Mapper, was used for specific pathway visualization (Kanehisa and Sato, 2020).

### Preparation of Sinisan

The mixture of Chaihu, Zhishi, Baishao, and Gancao (1:1:1:1) was purchased from Beijing Tongrentang (Beijing, China) and authenticated by our team. The mix was immersed in water for 30 min and extracted twice with boiling water as we previously mentioned (Wei et al., 2016). The extraction was filtered and concentrated in a rotary evaporator under reduced pressure. Ultimately, the dry powder was manufactured by a freeze dryer at a relatively low temperature condition (−80°C).

### UPLC-ESI-MS/MS Analysis

To confirm the important bioactive compounds in SNS, the SNS samples were analyzed using a UPLC-ESI-MS/MS system (UPLC, SHIMADZU Nexera X2; MS, Applied Biosystems 6500 Q TRAP). An Agilent SB-C18 column (1.8 µm, 2.1 mm*100 mm) was used. The mobile phase was pure water with 0.1% formic acid (A) and acetonitrile with 0.1% formic acid (B). Sample measurements were performed with a gradient program that employed the starting conditions of 95% A, 5% B. Within 9 min, a linear gradient to 5% A, 95% B was programmed, and a composition of 5% A, 95% B was kept for 1 min. Subsequently, a composition of 95% A, 5% B was adjusted within 1.10 min and kept for 2.9 min. The flow rate was 0.35 ml/min, and 2 μl of the filtrate was injected into the system for analysis. The ESI source operation parameters were as follows: ion source, turbo spray; source temperature 550°C; ion spray voltage (IS) 5500 V (positive ion mode)/−4500 V (negative ion mode); ion source gas I (GSI), gas II(GSII), and curtain gas (CUR) set at 50, 60, and 25.0 psi, respectively; and the collision-activated dissociation (CAD) was high. Instrument tuning and mass calibration were performed with 10 and 100 μmol/L polypropylene glycol solutions in QQQ and LIT modes, respectively. QQQ scans were acquired as MRM experiments with collision gas (nitrogen) set to medium. DP and CE for individual MRM transitions were done with further DP and CE optimization. A specific set of MRM transitions were monitored for each period according to the metabolites eluted within this period.

### Animals and Treatments

All animal experiments were approved by the Institutional Animal Care and Use Committee of the Department of Laboratory Animal Sciences, Capital Medical University. Animal Experimental Ethics number (AEEI-2020–165). Twenty-seven male Wistar rats (280–320 g) were purchased from Charles River Inc., (Vital River Ltd., Beijing, China) and kept at the SPF animal room of the Department of Laboratory Animal Sciences, Capital Medical University. All rats were randomly distributed into three groups after 1 week of acclimatization and were fed either standard chow (Chow group) or high fat diet (HFD and SNS group). After 4 weeks of feeding, rats in the SNS group (n = 9) were intragastrically administered with SNS exact per day (0.368 g/kg), and the Chow group (n = 9) and HFD group (n = 9) were given the same volume of distilled water to mimic the effects of oral gavage administration while remaining on the previous diet, respectively, for another 4 weeks. The dose of SNS was determined based on the body surface area index between humans and rats (Nair et al., 2018).

### Sample Collection

At the end of 8 weeks, the final body weights of rats were recorded before sacrificing. After anesthetized with pentobarbital sodium (25 mg/kg; IP), blood was obtained from the abdominal aorta and separated by centrifugation (3,000 rpm, 15 min) for serum collection after being kept at room temperature for 30 min, and the serum was collected from the supernatant of blood and stored at −80°C for the further determination. The liver was quickly removed, weighed, and thoroughly washed with phosphate buffer saline (PBS). A portion of the liver was stored separately in a 4% paraformaldehyde for histopathological examination. The rest of the liver was snap-frozen using liquid nitrogen for further investigation.

### Biochemical Indicators of Hepatic Function

The serum alanine aminotransferase (ALT), aspartate aminotransferase (AST), free fatty acids (FFA), total triglyceride (TG), total cholesterol (TC), low-density lipoprotein cholesterol (LDL-C), and high-density lipoprotein cholesterol (HDL-C) were detected by a Fully Automatic Biochemical Analyzer (Beckman Coulter, Indianapolis, IN, United States) according to manufacturer’s protocol.

### Measurement of Serum and Liver Cytokines

Serum and liver levels of TNFα, IL6, and IL1β were detected using ELISA kits of TNFα, IL6, and IL1β, following the manufacturer’s instructions, respectively. Absorbance at 450 nm was measured using a microplate spectrophotometer (Thermo Scientific Multiskan GO). Total protein was used to normalize these cytokine levels in the liver.

### Measurement of Advanced Glycation End-Products and Receptor for Advanced Glycation End-Products

Serum and liver levels of AGEs and RAGE were detected using ELISA kits of AGEs and RAGE, following the manufacturer’s instructions, respectively. Absorbance at 450 nm was measured using a microplate spectrophotometer (Thermo Scientific Multiskan GO). Total protein was used to normalize these levels in the liver.

### Histological Examination

The liver was immediately fixed in 4% paraformaldehyde overnight at room temperature, washed with ddH_2_O, rehydrated with gradient ethanol solutions, and embedded in paraffin for Hematoxylin–Eosin (H&E) staining. For the detection of lipids, frozen liver sections were stained with Oil Red O.

### Triglyceride and Cholesterol Evaluation in Liver

Liver samples of appropriate weight were homogenized with RIPA lysis buffer; homogenate was collected to assay intrahepatic triglyceride and cholesterol using commercial kits (Applygen, China). Triglyceride and cholesterol values were normalized by total protein contents.

### Immunoblotting Analysis

The liver tissues were homogenized with lysis buffer (Applygen, China) according to the manufacturer’s protocol. The protein extracts were separated by 8% SDS-PAGE electrophoresis and electro-transferred to PVDF membranes (Millipore, MA, United States). After blocking with 5% BSA, the membranes were incubated overnight at 4°C with specific primary antibodies against Phospho-JAK2 (Cell Signaling Technology, Danvers, MA, United States), Phospho-STAT3 (Cell Signaling Technology, Danvers, MA, United States), and β-actin (Cell Signaling Technology, Danvers, MA, United States) separately. Following four washes with Tris-buffered saline tween-20 (TBST), the membranes were incubated with the corresponding secondary antibodies as Goat Anti-Rabbit IgG (Lablead, China) and for 1 h at room temperature. Membranes were then washed four times with TBST, and the detection was carried out with an electrochemiluminescence (ECL) reagent (Applygen, China). The blot was imaged using EvolutionCapt FX6 and subjected to quantification analysis using Image J software. The results are expressed as the ratio of the relative intensity of the target proteins to that of the internal standard. JAK2 and STAT3 blots were performed by stripping and re-probing the blots with corresponding antibodies, respectively (Cell Signaling Technology, Danvers, MA, United States).

### Statistical Analysis

All statistical analyses were performed using one-way ANOVA followed by Dunnett’s Multiple Comparison Test, and all the bar plots were generated by GraphPad Prism 9 software; the data were expressed in terms of mean ± standard division (SD). Statistical significance was set at *p* < 0.05.

## Results

### Compounds and Targets of Sinisan

**FIGURE 2:**
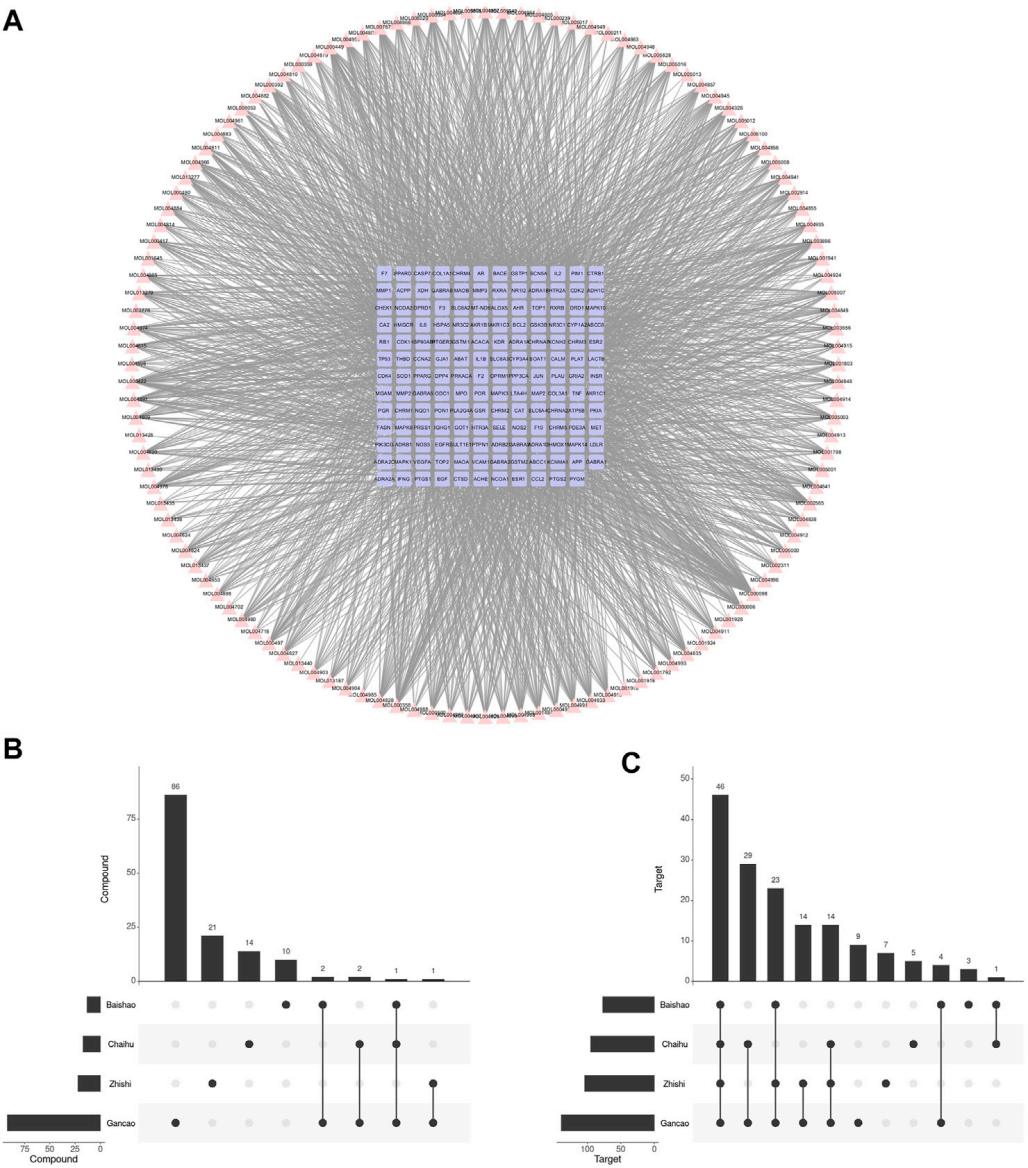
Compounds in SNS and their corresponding targets. **(A)** Compound-target network of SNS. **(B)** The SNS compound amount in each botanical drug. **(C)** The SNS target amount in each botanical drug.

After ADME screening, 137 unduplicated compounds and 155 unduplicated targets were obtained, which included 2,670 compound-target relationships (Figure 2A). Among four botanical drugs of Chaihu, Baishao, Zhishi, and Gancao, 17, 13, 22, and 92 compounds, respectively, were obtained (Figure 2B), which corresponded to 95, 77, 104, and 139 targets (Figure 2C). We found a potential synergistic effect of these four botanical drugs in the target level without many overlaps in the compound level. Gancao was the botanical drug that contributed to the highest proportion of collected compounds and targets.

### Relationship Between Sinisan Targets and Non-Alcoholic Fatty Liver Disease Genes

**FIGURE 3:**
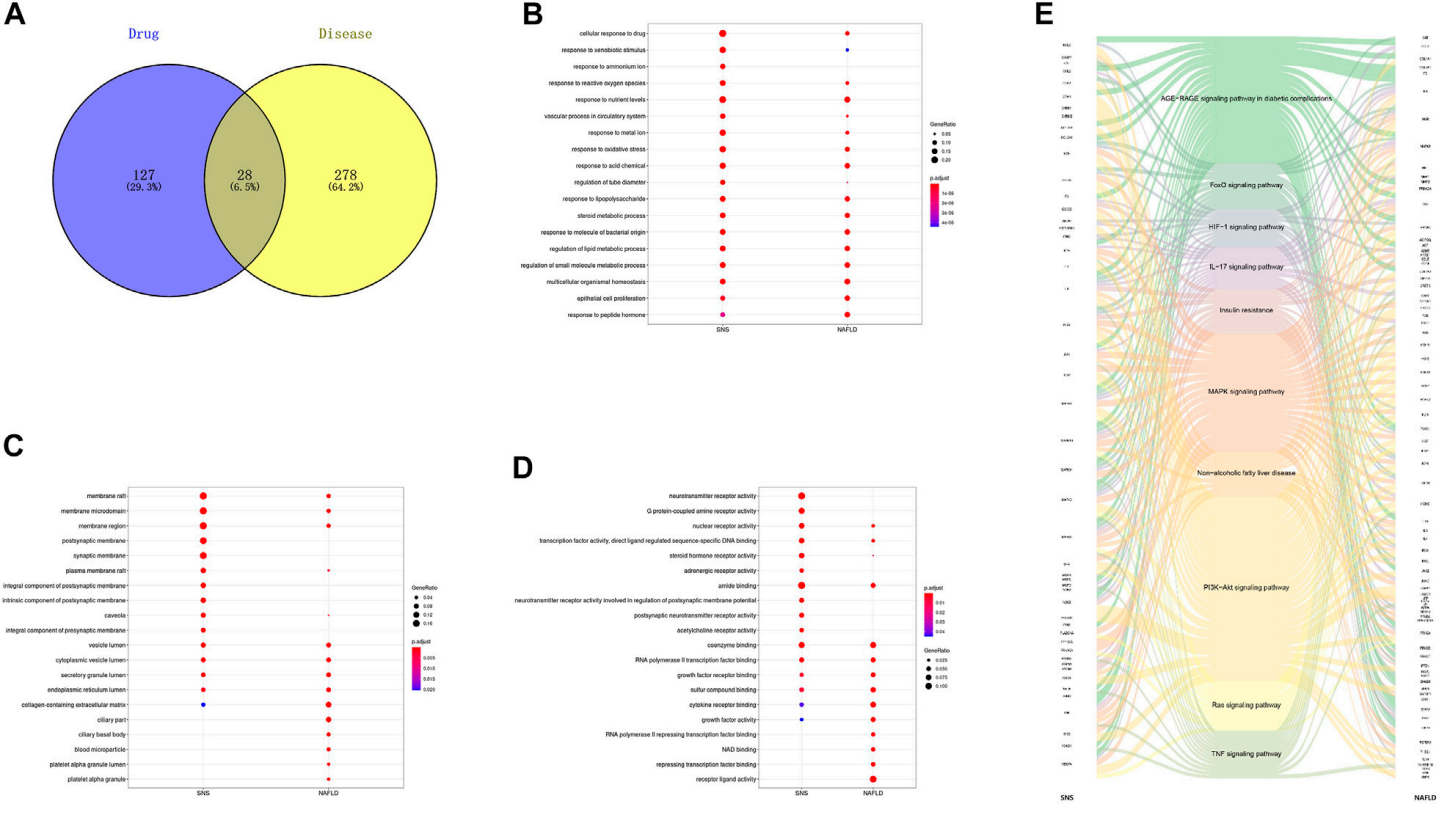
Overlapped terms-based analysis. **(A)** Veen diagram of compound targets of SNS and NAFLD-related targets. Overlapped gene ontology (GO) and Kyoto Encyclopedia of Genes and Genomes (KEGG) terms of SNS and NAFLD: **(B)** biological processes (BPs), **(C)** cellular components (CCs), **(D)** molecular functions (MFs), and **(E)** KEGG pathways.

After screening all of the compounds and targets, we want to recognize the potential corresponding compounds and targets of SNS anti-NAFLD effect. And we gathered genes related to NAFLD by database mining and removed predicted ones. As shown in Figure 3A, only 28 genes were overlapped between SNS genes and NAFLD genes. GO enrichment indicated that SNS targets and NAFLD genes could be enriched in the same GO terms with statistically significant difference (Figures 3B–D). These overlapped terms may involve lipid metabolism, oxidative stress, inflammatory progress, and transcription. After KEGG enrichment of each gene set, 83 KEGG signals were overlapped. Figure 3E exhibited a complex relationship among SNS targets, NAFLD genes, and representative KEGG signals. These results showed similar terms enriched with SNS targets and NAFLD genes, indicating potential therapeutic effects of SNS against NAFLD.

### Overlapped Gene-Based Enrichment Analysis

**FIGURE 4:**
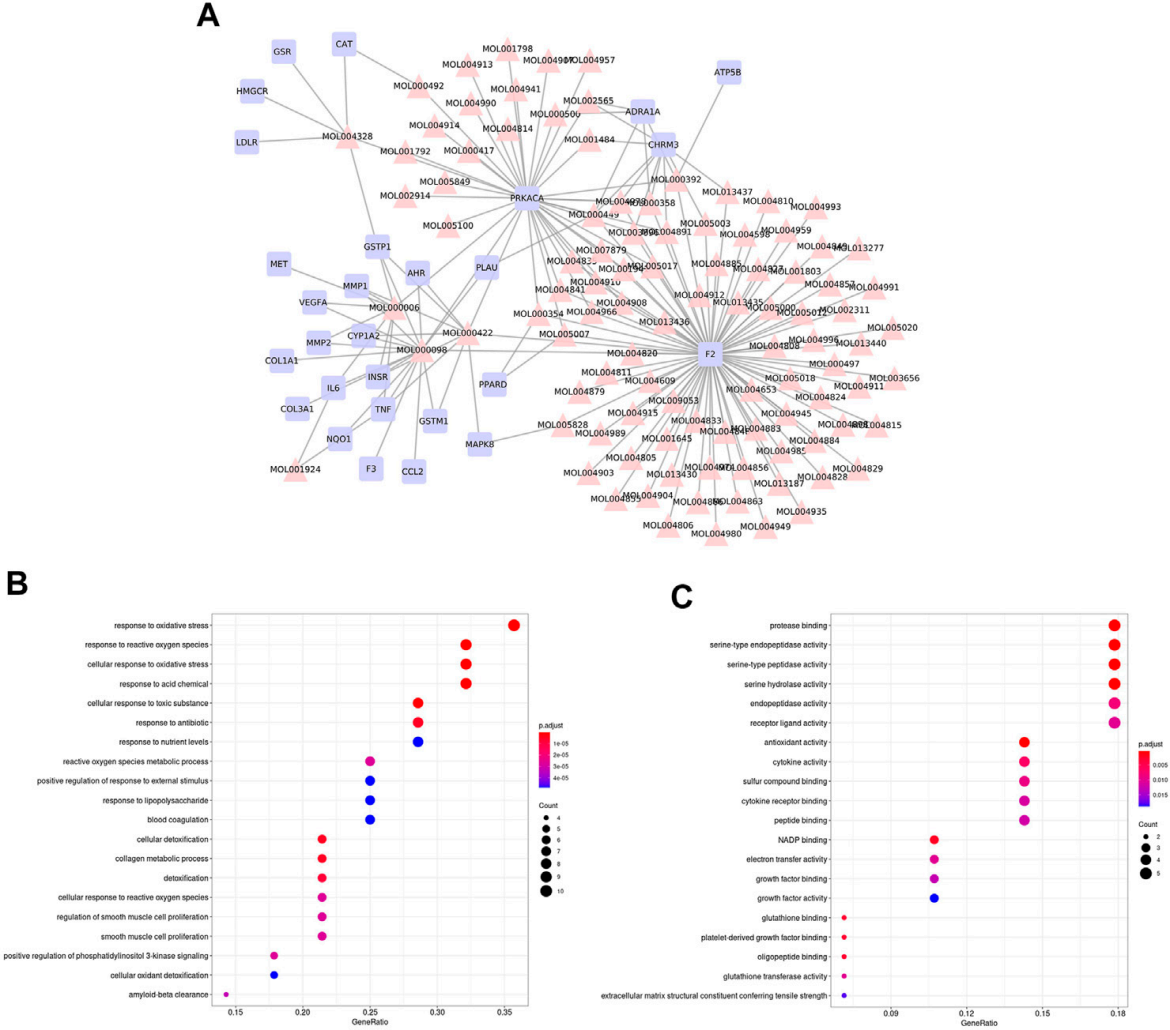
Overlapped genes-based analysis. **(A)** Compound-target network of SNS against NAFLD. Gene ontology (GO) enrichment analysis of overlapped genes: **(B)** biological processes (BPs) and **(C)** molecular functions (MFs).

We firstly constructed a compound-target network based on overlapped genes between SNS targets and NAFLD genes (Figure 4A). GO enrichment of these overlapped genes indicated the potential mechanisms of SNS against NAFLD related to response to oxidative stress, response to lipopolysaccharide (LPS), phosphatidylinositol 3-kinase (PI3K) signaling, cytokine activity, and antioxidant activity (Figures 4B,C).

### Protein-Protein Interaction Network-Based Proximity Analysis

According to previously published articles, disease genes tended to form a neighborhood in the human protein interactome, rather than being separate randomly (Cheng et al., 2019). And if a drug was effective for a disease, corresponding targets should be within or in the immediate vicinity of the corresponding disease module in the human protein interactome, indicating the role of neighborhood genes of disease genes in drug discovery. Our data showed the mean shortest path length between genes of SNS and NAFLD was 1.05, and the Z score was −9.51, indicating a significant difference between the mean shortest path lengths of SNS-NAFLD and random ones.

### Protein-Protein Interaction Network-Based Cluster Analysis

**FIGURE 5:**
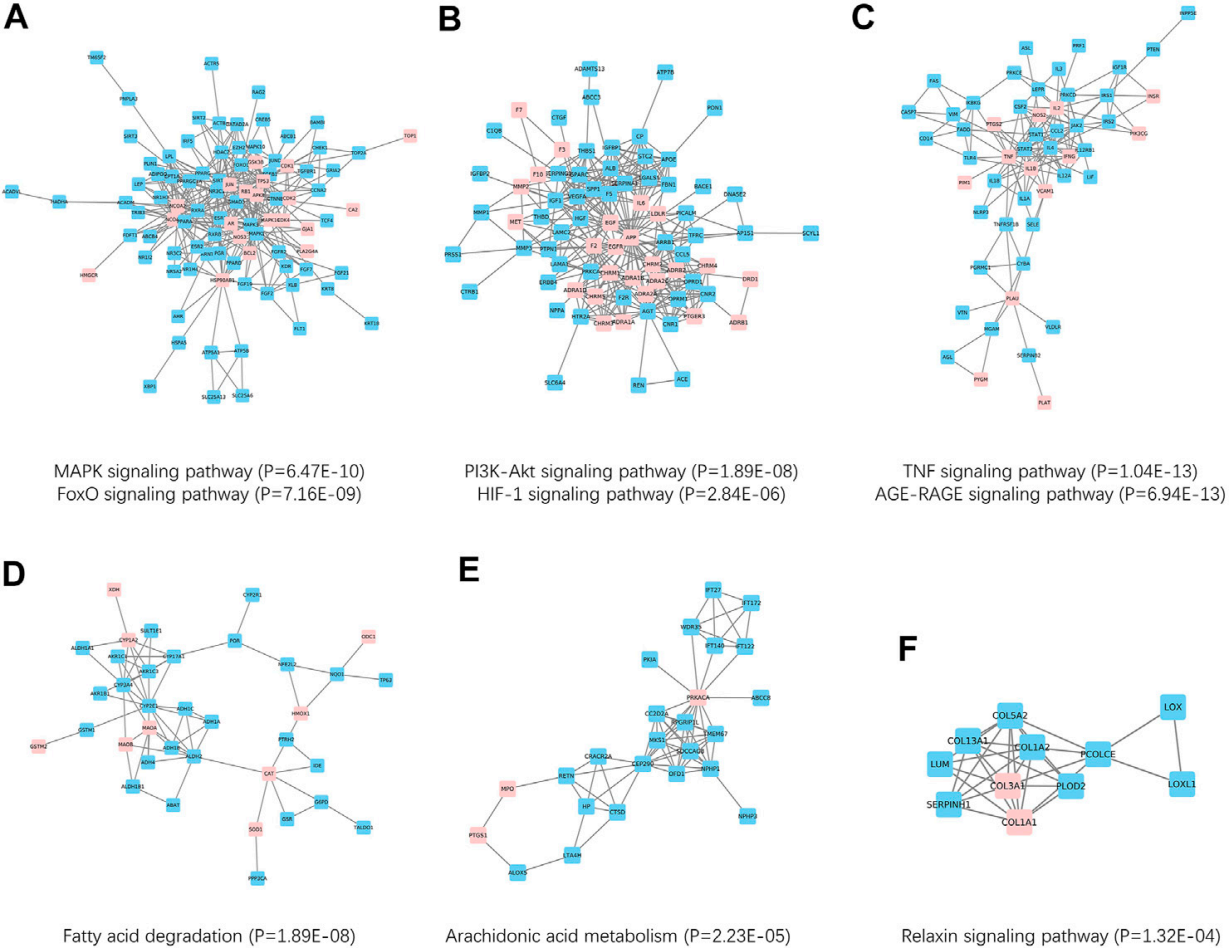
Network-based clusters identification and Kyoto Encyclopedia of Genes and Genomes (KEGG) enrichment. **(A)** Cluster 1 was related to MAPK and FoxO pathways. **(B)** Cluster 2 was related to PI3K-Akt and HIF-1 pathways. **(C)** Cluster 3 was related to TNF and AGE-RAGE pathways. **(D)** Cluster 4 was related to the fatty acid degradation pathway. **(E)** Cluster 5 was related to the arachidonic acid metabolism pathway. **(F)** Cluster 6 was related to the relaxin pathway.

The above results indicated a close relationship between SNS targets and NAFLD genes. Therefore, we hypothesized that SNS targets and NAFLD genes could form a network in the human protein interactome. To obtain data of high quality, a sub-PPI network with 1425 PPI relationships was grabbed from Snet. Further, Figure 5 A-F showed six PPI clusters identified, containing both SNS targets (pink) and NAFLD genes (blue). Cluster 1 is related to the MAPK signaling pathway and FoxO signaling pathway, cluster 2 is related to the PI3K-Akt signaling pathway and HIF-1 signaling pathway, cluster 3 is related to TNF signaling pathway and AGE-RAGE signaling pathway, cluster 4 is related to Fatty acid degradation, cluster 5 is related to Arachidonic acid metabolism, and cluster 6 is related to relaxin signaling pathway.

### Protein-Protein Interaction Network-Based Hub Genes Identification

Although proximity analysis evaluated the efficacy of drug targets on disease genes, it was no help in hub gene identification. Therefore, we needed a ranking algorithm called RWR. As shown in Table 1, the top 10 genes were identified as hub genes by executing the RWR algorithm on the above sub-PPI network, including STAT3, CTNNB1, MAPK1, MAPK3, AGT, NQO1, TOP2A, FDFT1, ALDH4A1, and KCNH2 (Table 1).

### Screening of Bioactive Compounds by UPLC-ESI-MS/MS Analysis

The present approach identified 58 bioactive compounds in SNS (Table 2), and some important bioactive compounds in the SNS compound-target network were identified, such as quercetin, kaempferol, medicarpin, luteolin, tetramethoxyluteolin, formononetin, isorhamnetin, naringenin, vestitol, and licochalcone A.

### Sinisan Attenuated High Fat Diet-Induced Hyperlipidemia

**FIGURE 6:**
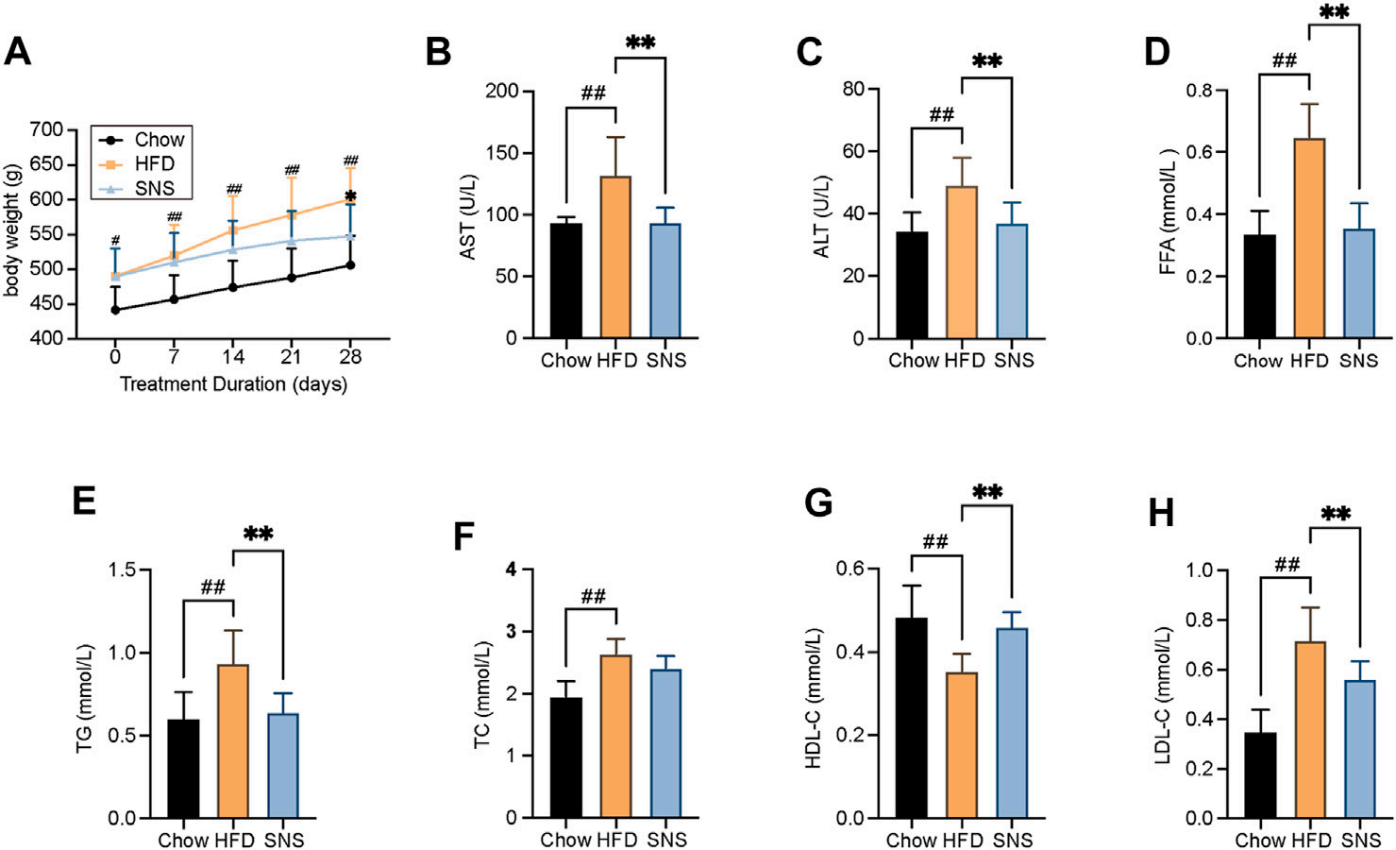
Body weight and biochemical assays results after SNS treatment for rat NAFLD model. **(A)** Body weight measurement. **(B)** Serum aspartate aminotransferase (AST) levels. **(C)** Serum alanine aminotransferase (ALT) levels. **(D)** Serum free fatty acid (FFA) levels. **(E)** Serum total triglyceride (TG) levels. **(F)** Serum total cholesterol (TC) levels. **(G)** Serum high-density lipoprotein cholesterol (HDL-C) levels. **(H)** Serum low-density lipoprotein cholesterol (LDL-C) levels. Data are shown as mean ± SD (n = 9). ^#^ *p* < 0.05, ^##^ *p* < 0.01, ^###^ *p* < 0.001 when compared with chow group. *p < 0.05, **p < 0.01, ***p < 0.001 when compared with HFD group.

To evaluate the therapeutic effect of SNS, a high fat diet- (HFD-) induced NAFLD rat model was used. As expected in the NAFLD model, HFD-treated rats exhibited an increased body weight. Compared with the HFD group, SNS treated rats showed significantly lowered body weights after a 4-week treatment (Figure 6A). The transaminase levels were assessed to evaluate whether SNS made improvements on liver function of NAFLD rats. The serum levels of AST and ALT were increased significantly in the HFD group compared with the Chow group, and these rises were reduced significantly by the SNS treatment compared with the HFD group (Figures 6B,C). It showed that levels of FFA were elevated in HFD rats, which manifests as metabolic disorders; however, it could be significantly alleviated after SNS intervention (Figure 6D). In addition, compared to the HFD group, SNS treatment influenced body lipid metabolism, including significantly decreasing the levels of serum TG and LDL-C (Figures 6E,H) and significantly increasing the levels of HDL-C (Figure 6G). SNS was also observed to decrease the levels of serum TC without significant difference (Figure 6F). These results indicated a therapeutic effect of SNS on HFD-induced hyperlipidemia.

### Sinisan Reduced High Fat Diet-Induced Hepatic Steatosis

**FIGURE 7:**
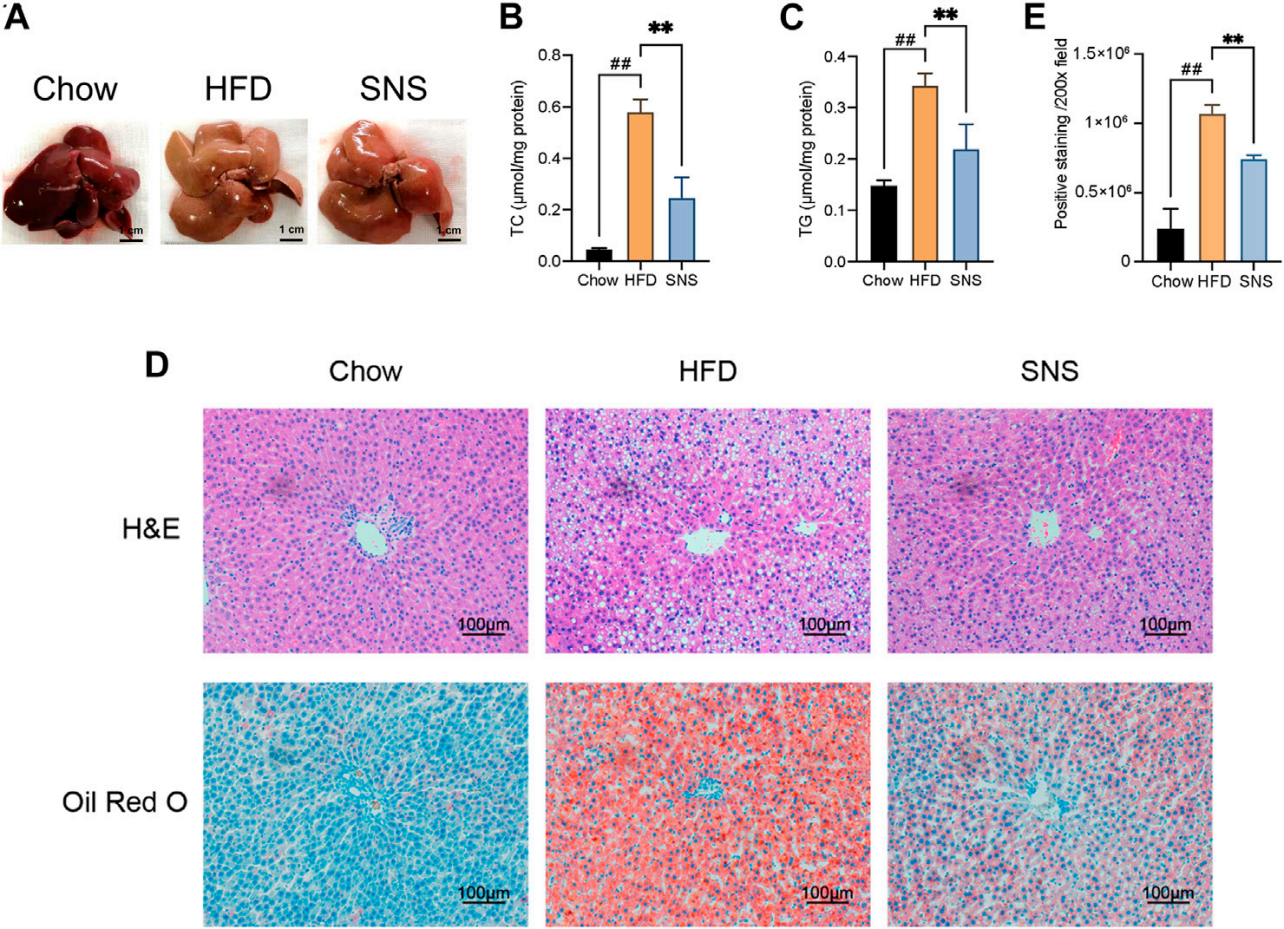
Sinisan reduced liver lipid accumulation. **(A)** Morphology of the liver in each group. **(B)** Liver total cholesterol (TC) levels normalized by total protein. **(C)** Liver total triglyceride (TG) levels normalized by total protein. **(D)**. HE-stained and Oil Red O-stained liver tissue. **(E)** Relative evaluation of Oil Red O-staining. Three biological replicates were performed for each study. ^#^ *p* < 0.05, ^##^ *p* < 0.01, ^###^ *p* < 0.001 when compared with the Chow group, *p < 0.05, **p < 0.01, ***p < 0.001 when compared with HFD group.

As shown in Figure 7A, in contrast to normal diet rats, HFD-fed rats developed a liver enlargement and discoloration, which were partially recovered with SNS treatment. Markedly, HFD-induced accumulation of lipid droplets in the liver was reduced by SNS, as evidenced by hepatic concentration of TC, TG (Figures 7B,C) and pathological staining (Figure 7D, representative histological liver H&E and Oil Red O staining). H&E staining revealed inflammatory cells and numerous lipid droplets in the livers of NAFLD rats, indicating inflammation and hepatocyte steatosis in the liver. SNS treatments showed less inflammation and hepatocyte steatosis than those of the NAFLD group. Oil Red O staining showed that there were many deposited lipid droplets in the HFD group. Compared with the HFD group, the lipid droplet quantity was lower in the SNS group (Figure 7E).

### Sinisan Ameliorated High Fat Diet-Induced Hepatic Inflammation

**FIGURE 8:**
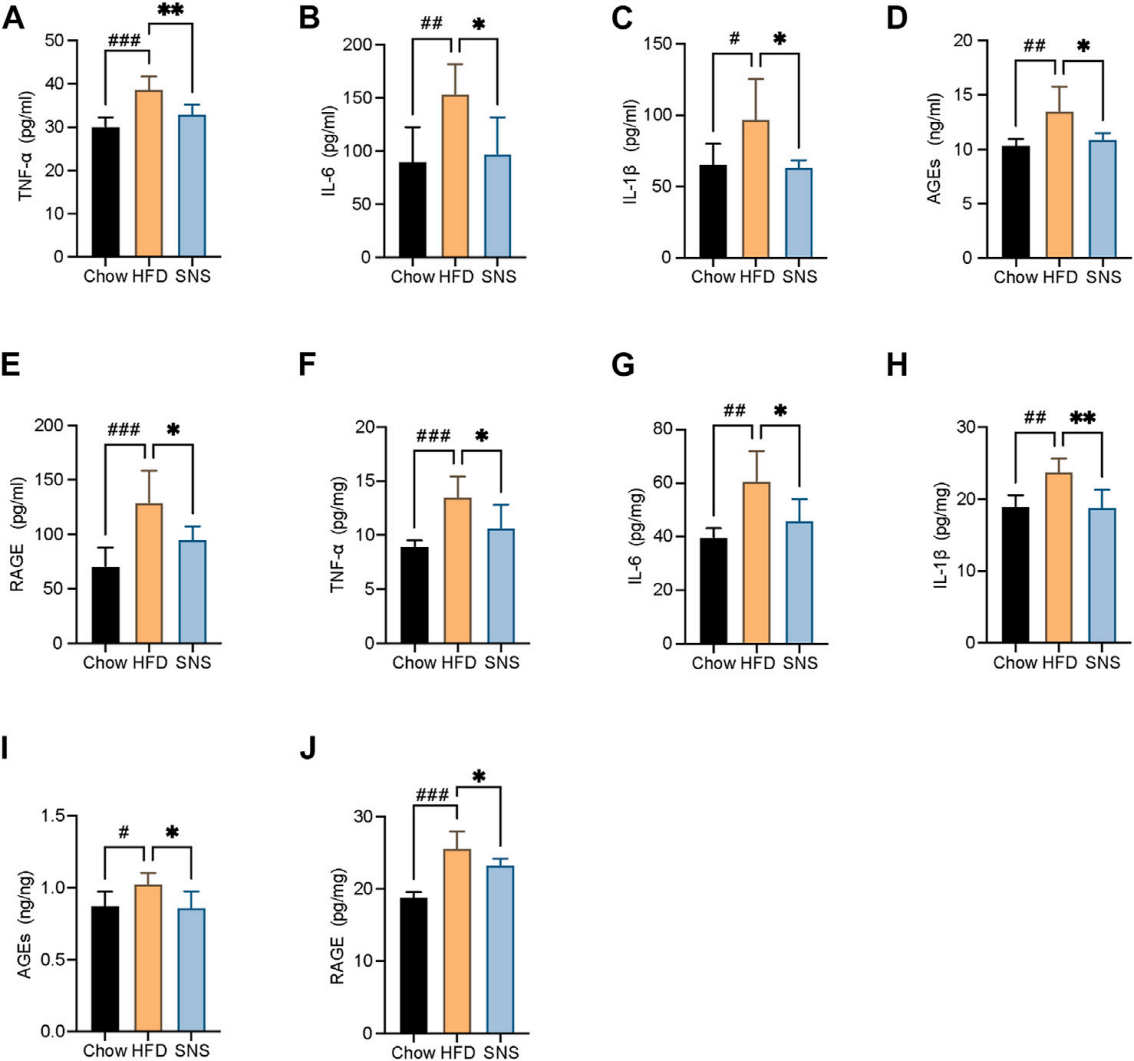
SNS reduced liver pro-inflammatory cytokines, advanced glycation end-products (AGEs), and its receptor. **(A)** Serum levels of tumor necrosis factor alpha (TNFα). **(B)** Serum levels of interleukin 6 (IL6). **(C)** Serum levels of interleukin 1β (IL1β). **(D)** Serum levels of AGEs. **(E)** Serum levels of receptor for advanced glycation end-products (RAGE). **(F)** Liver levels of TNFα. **(G)** Liver levels of IL6. **(H)** Liver levels of IL1β. **(I)** Liver levels of AGEs. **(J)** Liver levels of receptor for RAGE. Data are shown as mean ± SD (n = 6). ^#^ *p* < 0.05, ^##^ *p* < 0.01, ^###^ *p* < 0.001 when compared with the Chow group, *p < 0.05, **p < 0.01, ***p < 0.001 when compared with the HFD group.

To assess the extent of inflammatory, several inflammatory cytokines, AGEs, and RAGE in serum and liver were measured (Figures 8A–J). As a result, the HFD group displayed markedly higher expressions of IL6, IL1β, TNFα, AGEs, and RAGE compared with the Chow group. However, they were decreased in SNS-intervened rats compared with those in the HFD group, suggesting that SNS had an inhibitory effect on hepatic inflammation in NAFLD rats.

### Sinisan Inhibited JAK2/Signal Transducer and Activator of Transcription 3 Phosphorylation

**FIGURE 9:**
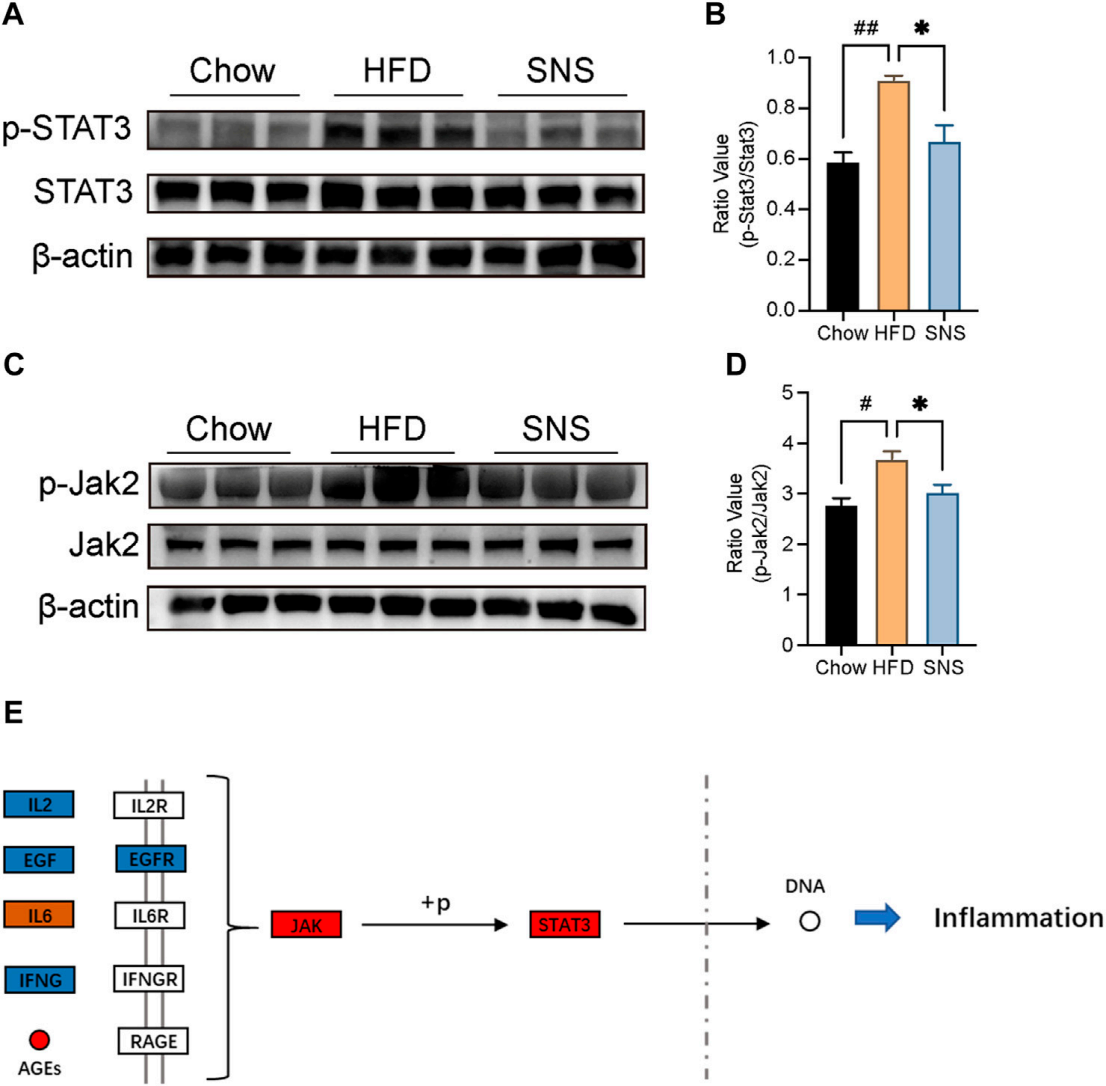
SNS inhibited JAK2/STAT3 signal against NAFLD. **(A)** Expressions of signal transducer and activator of transcription (STAT3) and phosphorylated STAT3 (p-STAT3) were determined by Western blotting. **(B)** Quantitative analysis of p-STAT3:STAT3 expression ratio. **(C)** Expressions of Janus Kinase 2 (JAK2) and phosphorylated JAK2 (*p*-JAK2) were determined by Western blotting. **(D)** Quantitative analysis of *p*-JAK2:JAK2 expression ratio. Data are shown as mean ± SD (n = 3). ^#^ *p* < 0.05, ^##^ *p* < 0.01, ^###^ *p* < 0.001 when compared with the Chow group, *p < 0.05, **p < 0.01, ***p < 0.001 when compared with HFD group.

To investigate the potential of SNS’s effect on the STAT3 signal in the NAFLD rat model, immunoblot analysis was performed for JAK2 and STAT3 phosphorylation (Figures 9A–D). It was demonstrated that expression of JAK2 and STAT3 phosphorylation was enhanced in NAFLD rats, while SNS reversed this activation to some extent. Therefore, we suggested that the inhibition of the JAK2/STAT3 signal may be the potential mechanism of SNS treating NAFLD (Figure 9E).

## Discussion

SNS has been widely applied in the treatment of chronic liver diseases in the clinic. As we previously mentioned, previous studies reported that SNS induced a decreased body weight and liver triglyceride accumulation in animal models (Cheng et al., 2017; Zhu et al., 2019). However, the underlying mechanisms of SNS against NAFLD are still difficult to understand, for it includes multiple compounds and multiple targets. In the present study, we modified our previously published network pharmacology approach (Ma et al., 2020) and used it to study the system-wide mechanism of SNS against NAFLD. This approach started from compounds screening, targets searching, and drug effect analyzing. Unlike previous studies, we hypothesized that SNS may influence NAFLD not only directly acting on the targets related to NAFLD, but also acting on the neighbors of NAFLD targets. Therefore, to evaluate the relationship between SNS targets and NAFLD targets, we used a well-performed network-based drug-disease proximity algorithm on the human interactome, which had an area under the receiver operating characteristic curve (AUC) of over 70% in a previous study (Cheng et al., 2018). After comparing with the reference distance distribution corresponding to the expected network topological distance between two randomly selected groups of proteins matched to size and degree as the NAFLD targets and SNS targets, the distance between the NAFLD targets and SNS targets was more close than the reference distance. These results indicated a therapeutic effect of SNS against NAFLD. To evaluate the importance of different targets, we used the RWR algorithm to sort the targets in a sub-PPI network only consisting of SNS targets and NAFLD targets. According to the RWR score, STAT3 was the most important target related to SNS targets. And most importantly, STAT3 played a core role in enriched KEGG pathways, including AGE-RAGE signal, FoxO signal, HIF-1 signal, and insulin resistance.

Our experimental data indicated that SNS treatment decreased Jak2/STAT3 activation, which could be activated by IL6 and AGEs. Previous studies showed that STAT3 played important roles in liver inflammation and interacted with multiple signals. GWAS study validated the genetic associations of STAT3 with the susceptibility to NAFLD and disease progression in the Asian population (Sookoian et al., 2008; Kumar et al., 2019). *In vivo* study indicated that STAT3 was related to lipid synthesis by modulating the expression of SREBP1 in a high-fat diet model (Zeng et al., 2017). *In vitro* obesity model showed that STAT3 knockdown significantly attenuated TG content and expression of SREBP1 in LO2 cells (Chen et al., 2018). Another function of the STAT3 signal was activating liver inflammation. It has been reported that hepatocyte-specific STAT3 knockout markedly inhibited liver inflammation compared with wild-type mice (Miller et al., 2011). STAT3 was considered highly interconnected with NFκB signal, a core transcription factor in diverse immune responses. Many inflammatory factors transcribed by NFκB, such as IL6, are important STAT3 activators. In some conditions, STAT3 could directly interact with NFκB and lead to constitutive NFκB activation and numerous inflammatory genes transcription (Yu et al., 2009). According to previous reports, IL6, IL1b, and CCL2 were upregulated by STAT3 activation (Yu et al., 2009). And these pro-inflammatory factors played important roles in the progression of NAFLD (Kazankov et al., 2019). Recent data showed that STAT3-related lysosomal membrane permeabilization promoted ferroptosis via CTSB (cathepsin B) (Zhou et al., 2020). These data supported the therapeutic effect of SNS against NAFLD.

Our network pharmacology identified three compounds in SNS related to STAT3 inhibition, including quercetin, paeoniflorin, and luteolin. Recent studies reported that quercetin interrupted the positive feedback loop between STAT3 and IL-6 and exhibited an anti-inflammatory phenotype (Granato et al., 2019). Luteolin was also found to decrease STAT-binding activity and markedly suppress STAT3 phosphorylation in a dose-dependent manner (Aziz et al., 2018). Except for these two flavonoids, paeoniflorin, a monoterpene glucoside, was proved to markedly decrease STAT3 activation in immune cells (Zhang and Wei, 2020). Future studies should test the relationship between STAT3 and these compounds' treatment in NAFLD models.

## Conclusion

In conclusion, in this study, we combined a PPI-network-based pharmacology analysis with biological validation to study the mechanism of the actions of SNS against NAFLD at the systemic level. We confirmed the anti-NAFLD effect of SNS was involved in multiple targets on Jak2/STAT3 signal pathway. In the future, other pathways or mechanisms predicted in this study should be further investigated.

## Data Availability

The original contribution presented in the study are included in the article/Supplementary Material; further inquiries can be directed to the corresponding authors.

## Abbreviations

AGEs: advanced glycation end-products; ALT: alanine aminotransferase; AST: aspartate aminotransferase; FFA: free fatty acid; HDL-C: high-density lipoprotein cholesterol; IL1β: interleukin 1β; IL6: interleukin 6; LDL-C: low-density lipoprotein cholesterol; NAFLD: non-alcoholic fatty liver disease; NFκB: nuclear factor kappa-B; NASH: non-alcoholic steatohepatitis; PI3K: phosphatidylinositol 3-kinase; PPI: protein-protein interaction; RAGE: receptor for advanced glycation end-products; RWR: random walk with restart; STAT3: signal transducer and activator of transcription; TC: total cholesterol Line; TG: total triglyceride; TNFα: tumor necrosis factor alpha.
